# A new scale assessing the stressors and rewards of children’s hospice work

**DOI:** 10.1186/s12904-023-01246-w

**Published:** 2023-09-13

**Authors:** Andrew Papworth, Andre Bedendo, Jo Taylor, Bryony Beresford, Suzanne Mukherjee, Lorna K Fraser, Lucy Ziegler

**Affiliations:** 1 School for Business and Society, University of York, York, YO10 5DD UK; 2 Department of Health Sciences, University of York, York, YO10 5DD UK; 3 Social Policy Research Unit, University of York, York, YO10 5DD UK; 4 Cicely Saunders Institute of Palliative Care, Policy and Rehabilitation, King’s College London, London, SE5 9RS UK; 5 School of Medicine, University of Leeds, Leeds, LS2 9JT UK

**Keywords:** Paediatric, Palliative, Hospice, Staff, Wellbeing, Scale

## Abstract

**Background:**

There is a workforce shortage in the children’s hospice sector, but there has been little research on the specific challenges of working in this setting and on how these challenges might be alleviated. To identify appropriate interventions to improve staff wellbeing, the drivers of wellbeing in children’s hospices need to be known and measured. This paper reports on the development of two measures, one for work-related rewards and one for work-related stressors, for use in children’s hospice care teams.

**Methods:**

A mixed-methods, four-stage study; the first three phases focused on the development of the scales, and the last stage focused on the validation of the scales. Participants of all stages were children’s hospice care team staff members in the UK. Stage 1: survey assessing the relevance and comprehensiveness of the original scale items (*N* = 60); Stages 2 (focus groups; *N* = 16) and 3 (cognitive interviews; *N* = 14) to assess content validity; Stage 4: UK-wide survey (*N* = 414) to validate the final version of the new, children’s hospice-specific scales using Rasch Analysis (RA) and Confirmatory Factor Analysis (CFA).

**Results:**

Due to poor fitting indices shown in the results from the RA, five items (out of 36) were removed from the new rewards scale used in the UK-wide survey and 20 (out of 62) were removed from the new stressors scale. CFA also supported the removal of the items and showed a one-factor structure for the rewards scale and a three-factor structure for the stressors scale were adequate—the sub-scales for the stressors scale related to caring for an ill or dying child (“Child” sub-scale), working with parents and families (“Parent” sub-scale), and stressors related to organisational factors, such as team conflict and workload (“Organisation” sub-scale).

**Conclusions:**

Both of the new scales showed good psychometric properties and can be useful in clinical settings and research to assess the perceived intensity of the work-related rewards and stressors for children’s hospice staff.

**Supplementary Information:**

The online version contains supplementary material available at 10.1186/s12904-023-01246-w.

## Background

Children’s hospices in the UK are key providers of care to children and young people with life-limiting conditions and their families [1, 2]. It is vital there are enough professionals with the skills and experience to meet the complex and holistic needs of seriously ill children and young people, and their families. Currently, there is a workforce crisis in children’s palliative care that is so severe it has led to some children’s hospices having to alter their service provision [3, 4]. The growing nursing vacancy rate in children’s hospices is higher than in the NHS, and posts are becoming increasingly difficult to fill [5]. The reasons for the workforce crisis are not fully understood but are likely to be multifaceted and complex. There is an expectation that caring for children and young people with life-limiting conditions is likely to be emotionally demanding; however there has been little research on the specific challenges of working in a children’s hospice setting [6]. The wellbeing of health professionals working in hospice settings has implications for staff retention and recruitment, and workplace absences, and influences the quality, cost and safety of patient care [7–9].

To identify appropriate interventions to improve staff wellbeing, the drivers of wellbeing in each setting need to be known and measured. Research has found that specific jobs have their own unique stressors and rewards, so it is essential to identify these [10]. One of the aims of the Staff Wellbeing in Children’s Hospices (SWiCH) study [11] was to address this evidence gap by developing and validating two new measures of work-related stressors and rewards experienced by staff working in children’s hospices. This paper reports on this process.

The children’s hospice scales were based on scales (herein the original scales) that were developed by the University of York (co-authors SM and BB) for measuring work-related stressors and rewards and validated in paediatric oncology multi-disciplinary teams [10, 12]. Although paediatric oncology care pathways are focused primarily on anti-cancer treatment with a largely curative intent, [13] there are many similarities with paediatric palliative care in terms of the stressors and rewards experienced by staff [14, 15]. In a report on the psychological wellbeing of staff at a UK children’s hospice [15], the original scales were reported as acceptable and relevant to children’s hospice staff, and feedback from staff indicated that minimal revisions to the validated scales were likely to be required.

This study applied the same transactional model of stress that was used to inform the development of the original scales [10]. This model states that nothing can be labelled as stressful unless it is appraised as such by an individual [16, 17]. In addition, this study drew on JD-R theory of working conditions [18, 19], which proposes that work-related stress is a response to an imbalance between the demands (such as work pressure and role ambiguity) on a worker and the resources (such as social support and autonomy) they have, or can mobilise, to meet those demands. While each occupation is different, the JD-R theory proposes staff wellbeing results from the interaction between these categories.

## Methods

**Fig. 1:**
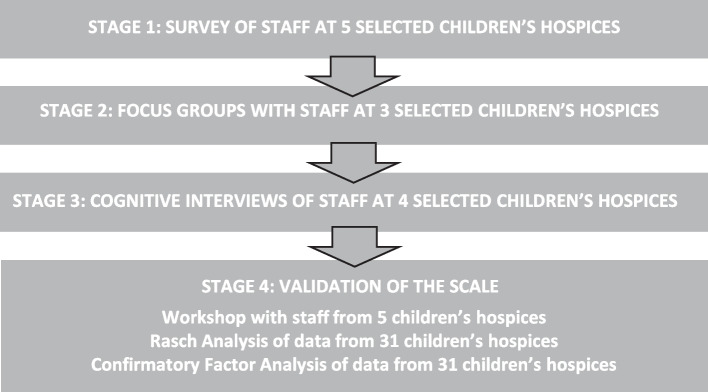
The development and validation of the children’s hospice stressors and rewards scales. Source: Authors

We used a rigorous, mixed methods exploratory design [20] comprising four phases (Fig. 1), with the first three phases focused on the development of the scale, and the last stage focused on its validation.

### The paediatric oncology scale

The two original scales are called the Work Rewards Scale-Paediatric Oncology (WRS-PO) and the Work Stressors Scale-Paediatric Oncology (WSS-PO), and contain 36 and 60 items respectively [21]. They are self-report measures of the frequency and intensity of work-related stressors and rewards experienced by an individual during the previous 6 months, which was found to be an acceptable length of time for individuals to be able to recall incidents and has been used for other similar scales [10, 22, 23]. The scales include items that are recognised as potential stressors and rewards across many occupational settings as well as items that are specific to working with children and young people who are seriously ill. Respondents are asked how often they have encountered the situation described by each item and how stressful or rewarding this situation is for them. Both measurements have three-point scales. Only the intensity measurement (how stressful or rewarding an item is perceived to be) is used to create the final scores. The frequency measurement (how often a situation is encountered) was included as it was found that when respondents reflected on this, they were more able to answer the intensity measurement. This format was retained for the new scales designed for children’s hospice staff.

### Recruitment

As there was a risk that the process of participating in the study might cause distress, or alert an individual to their need for support, staff were recruited via their hospice organisations for all four stages of the study. Hospices agreed to provide confidential support to participants if required. This information was detailed on the localised participant information sheets for each hospice that took part.

### Stage 1: staff survey of selected UK children’s hospices

In order to obtain feedback on the relevance of all items to the children’s hospice setting and the comprehensiveness of the item list, we invited care team staff (all those with a caring role) from a sample of five children’s hospices to provide individual feedback on these scales (see Table 1 for demographic information for Stage 1). Participants were grouped into two broad subgroups based on whether the staff provided medical and/or nursing care(i), or not(ii):

i) Medical and Nursing: Medics (Consultants, GPs, Paediatricians) and Registered Nurses
ii) Other: Allied Health Professionals, Nursery Nurses, Psychologists, Other

**Table 1:** Sample characteristics (Stage 1, Survey)

	Total
	(*N* = 60)
**Hospice Type**	
Children’s hospice that is part of a larger organisation	8 (13.3%)
Children’s hospice connected to an adult hospice	14 (23.3%)
Standalone children’s hospice	38 (63.3%)
missing	0
**Respondent’s Role**	
Medical and Nursing	28 (46.7%)
Other	31 (51.7%)
missing	1 (1.7%)

The hospices were purposively sampled to ensure they were representative of the differences between hospices across the UK. Sampling characteristics included size, income, and whether they were part of a larger hospice group, connected to an adult hospice, or were a standalone hospice organisation. All care team staff in the selected hospices were invited to take part. Sixty staff members responded to the survey between June and August 2019.

Participants were asked to rate each item in the WSS-PO and WRS-PO scales according to its relevance to their experiences working as part of a care team in a children’s hospice. The scales were presented using the same format as the WSS-PO and WRS-PO, but the original scales’ response options (*how often* and *how rewarding*) were changed to allow an assessment of relevance (*relevant; relevant but needs re-wording; not relevant*). Space was left for participants to write in new children’s hospice-specific stressors and rewards that were not represented in the WSS-PO and WRS-PO scales. To maximise response rates, participants were given the option of completing the task online [24] or on paper. Each participant was asked (after completing the task) if they were willing to take part in a focus group or follow-up interview for Stages 2 and 3.

The responses were then aggregated for all participants (% relevant/not relevant/relevant but needs re-wording). JT and AP reviewed any items where over 15% of respondents indicated they needed to be removed or reworded, and conducted a content analysis of all the new items proposed by participants. This work produced version 1 of the new children’s hospice scales. These were called the Work Stressors Scale – Children’s Hospices (WSS-CH) and the Work Rewards Scale – Children’s Hospices (WRS-CH).

### Stage 2: focus groups

To refine version 1 of the WSS-CH and WRS-CH scales, JT and AP conducted focus groups with children’s hospice care team staff members in three hospices (one focus group in each hospice; 16 participants in total) between September and October 2019. These were designed to help assess and refine the content validity of the re-worded and new items, and identify any further missing items. Version 1 of the scales were provided to focus group participants in advance and they were asked to discuss the relevance and meaning of new and re-worded items, and to identify stressors and rewards that they felt were not currently represented in the measures.

The first two focus groups took place in hospices where some staff had already taken part in the survey administered in Stage 1 of the scale development. These two focus groups were audio recorded and transcribed intelligent verbatim by a professional transcription company and anonymised for analysis. JT and AP conducted a content analysis to identify distinct stressors and rewards that were not already included in the draft scales, and revised the wording of the new or re-worded items.

The new and revised scale items, forming version 2 of the WSS-CH and WRS-CH scales, were presented to the attendees of the third focus group, who discussed the new and revised items until no further revisions and additions were required. Staff from this hospice had not taken part in Stage 1. JT and AP conducted further content analysis of this focus group to produce version 3 of the scales.

### Stage 3: cognitive interviews

This stage was designed to confirm that the response format for version 3 of the scales was appropriate and to make a final assessment of their content validity and participant comprehension and acceptability. AP and JT conducted cognitive interviews with 14 staff members from four hospices between January and February 2020. The participants represented all staff groups and two of the included hospices had not participated at any stage of the survey development up to this point. The cognitive interviews used the ‘think-aloud’ technique with concurrent verbal probing [25, 26].

Data saturation was monitored to determine final sample size (i.e., when no new feedback was being obtained from subsequent participants, data collection stopped) [27]. Audio data were transcribed and extracted into a participant-by-item matrix using directed content analysis, before being synthesised into a further matrix summarising key findings across participants for each item. This stage produced version 4 of the scales, which were used in the national survey of UK children’s hospice staff (see Stage 4, below).

### Stage 4: validation of the draft scales

This stage was designed to determine which items in the draft stressors (WSS-CH) and rewards (WRS-CH) scales should be included in the final versions of the scale, and to assess the scaling properties of the measures. This was conducted as part of the UK-wide survey of children’s hospice staff that formed the data collection phase for Phase 2 of the SWiCH study [11]. Data collection took place between May and December 2020. The data collection period was extended because of the impact of the COVID-19 pandemic.

The UK-wide survey collected demographic information about the participants and contained a suite of measures related to psychological wellbeing. The findings from the analysis of this survey are presented elsewhere [28, 29]. One of the measures used was version 4 of the WSS-CH and WRS-CH scales. The responses participants gave to the HSE Management Standards Tool and the Maslach Burnout Inventory, which were included in the UK-wide survey, were used to assess the construct validity of the new children’s hospice scales using Spearman’s Rho. The survey was designed and administered using Qualtrics.

We included all employed members of the care team regardless of their professional or occupational background, provided their main role was to provide direct care to children and their families. Staff were invited to take part in the survey by the participating organisation. A participant information sheet was provided, which explained the voluntary nature of participation and the confidentiality and anonymity afforded to participants. To maximise response rates, we also incentivised staff using a prize draw with several prizes offered to reflect the size of the sample.

The response data were extracted from the Qualtrics website into Excel sheets and were then cleaned by the study team. The scale validation analysis procedure was based on that used in the original scales development study [10] and followed a two-step approach: 1) Rasch Analysis (RA); and 2) Confirmatory Factor Analysis (CFA).

The preliminary RA of the responses to the version 4 of the WSS-CH and WRS-CH scales, conducted by AP and AB, highlighted that there were several co-dependent items in the scales. To enhance our understanding of these co-dependent items, AP, LZ and AB invited care team staff (*n* = 5) from three hospices to an online (Zoom) workshop. No staff members from two of the three hospices had participated in any of the first three scale development stages (Stages 1–3), but some staff members from all three hospices had responded to the UK-wide survey. Each participant received a participant information sheet and confirmation of informed consent was conducted using an electronic form. The results of this workshop were used to inform discussions during the final RA and CFA analyses of the scale data collected in the UK-wide survey [30–32].

The final version of the RA was used to determine items that should be included in the final scales and used the Partial Credit Model approach [33]. The Model’s overall goodness-of-fit was tested using the Andersen's likelihood-ratio test [34], where a non-significant p-value suggests a good fit [35]. Reliability was tested using Cronbach’s alpha and aimed at values higher than 0.7 [36, 37]. Cronbach’s alpha is an equivalent measure of the RA’s Person Separation Index (PSI)—Cronbach’s alpha uses the raw value whereas the PSI uses the logit value [36, 37]. Unidimensionality was tested using the Martin-Löf test [38], and used the mean as split criterion to define the item groups. A non-significant p-value supports unidimensionality [36].

Infit (weighted by the distance between the person position and item difficulty) and outfit (an unweighted measure) mean squares and t-statistics were assessed to identify misfitting items [39]. Mean squares searched for values under 0.7 or over 1.3 and t-statistics for values smaller out of the ± 2 range [33, 35]. Items with an indication of misfit were reviewed and removed from the scale. Item exclusion occurred using a stepwise approach and subsequent models were reviewed for improved item fit.

RA of the WRS-CH considered the full scale (36 items) and WSS-CH used the three sub-scales (factors) [“Child” (22 items); “Parent” (18 items); and “Organisation” (22 items)] used in the original scales [21]. Item numbers and labels for WRS-CH are presented in Supplementary Tables 1 and 2 for WSS-CH, and new items are identified**.**

After RA, we performed a CFA using the final set of items suggested from the RA and considered the Diagonally Weighted Least Squares (DWLS) estimator. Again, we considered a single factor for the WRS-CH and three factors for the WSS-CH. After initial fitting, modification indices were used to check for additional paths that could improve the model [40]. As modification indices can be viewed as χ2 statistics with 1 df, a value of 10.83 or greater was used as indicative that the overall model fit could be significantly improved (*p* ≤ 0.001) [41]. Correlated errors with a modification index higher than 11 (rounded from 10.83) were checked for theoretical plausibility (e.g., items that are similarly worded) and models were updated accordingly and fit re-checked. 

As indication of good model fit, we considered a Root Mean Square Error of Approximation (RMSEA) of ≤ 0.07 [42], Standardized Root Mean Square Residual (SRMR) < 0.10 [43], Comparative Fit Index (CFI) ≥ 0.90 [44], and Tucker-Lewis index (TLI) ≥ 0.90 [45].

All analyses were performed using R v4.2.1 [46] and R Studio v2022.07.1 + 554 [47]. RA used packages *ltm v1.2–0* [48] and *eRm* v1.0–2 [49]. CFA used package *lavaan* [50]. Models were calculated using only those participants with complete data available (WRS-CH: N = 375 and WSS-CH: *N* = 310), which gave a sufficient sample size for the RA, with a 99% confidence of person estimation and ± 0.5 logits, [51] and CFA estimation parameters [52]. All analysis used a significance level of 5%.

## Results

### Development of the scales (Stages 1–3)

The results from Stages 1–3 of the study showed that most of the items used in the original (paediatric oncology) scales were determined by children’s hospice staff to be suitable for use in the children’s hospice scales. In total, 83% of the items in the WRS-PO were included in version 4 of the WRS-CH scale, and 84% of the items in the WSS-PO were included in version 4 of the WSS-CH scale, which was completed by staff during the UK-wide survey and validated in Stage 4. A full breakdown of the changes made is presented (Supplementary Tables 3 and 4).

The items that were removed from the paediatric oncology scales, or were reworded or newly introduced during the first three stages, revealed some key differences between the two settings. Firstly, in contrast to paediatric oncology staff, children’s hospice staff can find some elements of the death of a patient rewarding. This was demonstrated by the new or reworded items in version 4 of the WRS-CH scale such as, “Getting it right for a child when they die”, “Making a child comfortable at the end of life” and “Helping a family to make memories”. Secondly, the nature of the long-lasting relationships that children’s hospice staff sometimes develop with the families of children is seen with the creation of the following new items in the WRS-CH scale: “Empowering children and their families to make decisions about care” and “Supporting the family after the death of their child”. Thirdly, many of the children’s hospice staff said that parents were often the most knowledgeable person about their child’s care, and the following item from the WSS-PO was removed as a result: “Parents thinking they know better than me”. Finally, as most children cared for in hospices have life-limiting or life-threatening conditions, the item “Lots of very sick children on the ward at once”, was adjusted to “Lots of very complex children at the same time” for the WSS-CH scale.

### Validation of the scales (Stage 4)

#### Sample characteristics

Out of 583 staff who took part in the survey, 414 participants had complete data for the WRS-CH or WSS-CH scales. Table 2 presents the sample characteristics. Hospice types were similarly distributed, but children services connected to adult hospices were slightly more frequently reported (36.5%, *N* = 127). Participants were mostly female (95.7%, *N* = 396) and aged between 41–65 years (58.7%, *N* = 243). Almost a third of our sample (30.0%, *N* = 124) reported 15 years or more of experience working with life-limiting conditions and around two thirds worked as full-time staff (62.6%, *N* = 259). During the COVID pandemic, most participants went to work as normal (58.5%, *N* = 220), although qualitative content analysis of the free-text responses for this item determined that some of those who selected “working as normal” had been redeployed to community services, or to a connected adult hospice within the same organisation.

**Table 2:** Sample characteristics – Stage 4

	Total
	(*N* = 414)
**Hospice Type**	
Children part of an organisation	116 (33.3%)
Connected to adult hospice	127 (36.5%)
Standalone Children	105 (30.2%)
missing	66
**Gender**	
Female	396 (95.7%)
**Age**	
21–30	68 (16.4%)
31–40	98 (23.7%)
41–50	116 (28.0%)
51–65	127 (30.7%)
66 +	5 (1.2%)
**Professional**	
Medical and Nursing	232(56.0%)
Other	182(44.0%)
**Years of experience working with life-limiting conditions**	
Less than 1 year	22 (5.3%)
1–2 years	45 (10.9%)
3–5 years	69 (16.7%)
6–10 years	81 (19.6%)
11–15 years	73 (17.6%)
More than 15 years	124 (30.0%)
**Full or part time**	
Full time	259 (62.6%)
Part time	155 (37.4%)
**WRS-CH Raw score**	
M(SD)	60.5 (11.2)
Range	4.0—72.0
*missing*	*39*
**WRS-CH Scaled score**	
M(SD)	78.4 (15.7)
Range	18.6—100.0
*missing*	*39*
**WSS-CH Raw score**	
M(SD)	50.7 (25.6)
Range	0.0—123.0
missing	104
**WSS-CH Scaled score**	
M(SD)	56.3 (9.5)
Range	0.0—100.0
*missing*	*104*
**Working scheme during COVID**	
Going into work as normal	220 (58.5%)
Working from home	63 (16.8%)
Mix of home / normal workplace	74 (19.7%)
At home on furlough pay	19 (5.1%)
*missing*	*38*

### Rasch analysis

The initial RA was used to identify misfitting items. Overall model fit statistics for the WRS-CH scale suggested a good fit model, but overall goodness of fit for the three WSS-CH sub-scales suggested a poor fit (Supplementary Table 5). Next, we examined individual item fit statistics (Chi-squared, MSQ, and t statistics). Items that showed at least two estimates as distorting/degrading the measurement, or were less productive for the measurement system, were then reviewed by AB and AP against findings from Stages 2 and 3.

We re-ran the models excluding items with clear justification for removal. Further refits were performed by including additional items that had less justification for exclusion. Overall model fit parameters were checked on each iteration to ensure adequate fit. The final supported models are shown in Table 3. Item and person fit indicators are presented in Table 4 and showed that on average, included items had values within the expected ranges.

**Table 3:** Overall model fit statistics for Rasch analysis

	**Cronbach's alpha**	**Unidimensionality**		**Overall goodness-of-fit**	
		LR-test (Chi-squared df)	*p* value	LR-test (Chi-squared df)	*p* value
WRS-CH	0.94	265.63 (951)	1.00	24.57 (21)	0.266
WSS-CH – Child	0.94	339.23 (1279)	1.00	32.13 (25)	0.154
WSS-CH – Parent	0.91	210.89 (279)	0.999	30.02 (31)	0.516
WSS-CH – Organisational	0.89	145.16 (167)	0.888	34.95 (23)	0.053

Cronbach's alpha. Should be > 0.70 to be statistically reliable

Unidimensionality tested using Martin-Löf-Test. Non-significant p-value at the 5% level supports unidimensionality

Overall goodness-of-fit tested using Andersen's likelihood-ratio test. Non-significant *p*-value at the 5% level supports a good fit

*WRS-CH* Work Rewards Scale – Children’s Hospices

*WSS-CH* Work Stressors Scale – Children’s Hospices

**Table 4:** Overall item and person fit indicators for WRS-CH and WSS-CH scales

	Outfit MSQ—Mean(SD)	Infit MSQ—Mean(SD)	Outfit t—Mean(SD)	Infit t—Mean(SD)
WRS-CH				
* Item*	0.98(0.14)	0.98(0.10)	-0.05(0.98)	-0.18(1.08)
* Person*	0.98(0.41)	0.99(0.31)	-0.05(1.34)	-0.04(1.35)
WSS-CH				
*WSS-CH—Child*				
*Item*	0.95(0.11)	0.96(0.08)	-0.53(1.29)	-0.61(1.12)
* Person*	0.95(0.45)	0.97(0.43)	-0.26(1.49)	-0.24(1.53)
* WSS-CH—Parent*				
* Item*	0.95(0.09)	0.94(0.05)	-0.58(1.08)	-0.78(0.69)
* Person*	0.95(0.51)	0.96(0.45)	-0.21(1.29)	-0.19(1.3)
* WSS-CH—Organisational*				
* Item*	0.94(0.11)	0.96(0.08)	-0.66(1.3)	-0.65(1.17)
* Person*	0.94(0.40)	0.95(0.37)	-0.16(1.25)	-0.16(1.29)

*WRS-CH* Work Rewards Scale – Children’s Hospices

*WSS-CH* Work Stressors Scale – Children’s Hospices

The final models removed items 7, 9, 10, 13 and 22 from the WRS-CH scale. For the WSS-CH scale, we removed items 1, 13, 21, 23 and 33 from the Child sub-scale, items 27, 40, 49, 55 and 58 from the Parent sub-scale and items 8, 25, 26, 35, 42, 44, 50, 51, 54 and 59 from the Organisational sub-scale. Finally, item fits were again examined (Tables 5 and Table 6). The full-text description for all items is included in the Supplementary Material.

**Table 5:** Fit statistics for the WRS-CH scale items after removing misfitting items

	Chi-squared	df	*p*-value	Outfit MSQ	Infit MSQ	Outfit t	Infit t	Discrimination
Item 01	302.3	322	0.778	0.94	0.89	-0.30	-1.10	0.59
Item 02	328.2	322	0.394	1.02	0.94	0.15	-0.54	0.56
Item 03	332.1	322	0.337	1.03	1.13	0.23	1.39	0.44
Item 04	331.4	322	0.347	1.03	1.02	0.24	0.24	0.53
Item 05	240.1	322	1.000	0.74	0.90	-1.90	-1.18	0.59
Item 06	383.5	322	0.010	1.19	0.94	0.84	-0.47	0.52
Item 08	320.2	322	0.517	0.99	0.97	-0.05	-0.36	0.55
Item 11	324.1	322	0.457	1.00	0.90	0.07	-1.06	0.59
Item 12	383.7	322	0.010	1.19	1.15	2.09	2.03	0.39
Item 14	377.1	322	0.019	1.17	1.09	1.89	1.19	0.45
Item 15	247.0	322	0.999	0.77	0.85	-1.80	-1.76	0.64
Item 16	355.5	322	0.096	1.10	1.09	0.75	0.96	0.49
Item 17	272.1	322	0.980	0.84	0.87	-1.15	-1.54	0.63
Item 18	377.4	322	0.018	1.17	1.09	0.72	0.81	0.53
Item 19	342.7	322	0.205	1.06	0.98	0.46	-0.19	0.56
Item 20	364.5	322	0.051	1.13	1.04	0.85	0.41	0.51
Item 21	292.0	322	0.884	0.90	0.90	-0.78	-1.22	0.61
Item 23	259.2	322	0.996	0.80	0.89	-0.89	-0.93	0.60
Item 24	307.0	322	0.717	0.95	0.92	-0.30	-0.90	0.59
Item 25	319.5	322	0.529	0.99	0.97	-0.06	-0.42	0.56
Item 26	354.6	322	0.102	1.10	1.11	0.89	1.36	0.47
Item 27	333.9	322	0.312	1.03	1.06	0.35	0.82	0.48
Item 28	344.1	322	0.190	1.07	0.93	0.48	-0.76	0.59
Item 29	309.8	322	0.677	0.96	1.08	-0.22	0.89	0.52
Item 30	342.2	322	0.210	1.06	1.01	0.55	0.19	0.54
Item 31	288.8	322	0.908	0.89	0.84	-0.60	-1.66	0.64
Item 32	330.9	322	0.355	1.02	1.09	0.18	0.82	0.55
Item 33	292.7	322	0.878	0.91	1.05	-0.62	0.57	0.51
Item 34	312.2	322	0.641	0.97	1.03	-0.10	0.26	0.49
Item 35	247.7	322	0.999	0.77	0.83	-1.71	-1.90	0.64
Item 36	200.6	322	1.000	0.62	0.81	-1.80	-1.58	0.65

**Table 6:** Fit statistics for the WRS-CH scale items after removing misfitting items

	Chi-squared	df	*p*-value	Outfit MSQ	Infit MSQ	Outfit t	Infit t	Discrimination
**WSS-CH—Child**								
Item 02	338.1	348	0.638	0.97	0.97	-0.38	-0.36	0.57
Item 05	360.4	348	0.312	1.03	0.97	0.41	-0.39	0.57
Item 07	290.2	348	0.989	0.83	0.84	-2.36	-2.33	0.64
Item 09	349.6	348	0.466	1.00	1.00	0.05	0.04	0.52
Item 11	295.0	348	0.982	0.85	0.86	-2.10	-2.06	0.63
Item 12	358.8	348	0.333	1.03	1.04	0.39	0.63	0.55
Item 16	371.8	348	0.182	1.07	1.08	0.75	1.15	0.51
Item 17	330.8	348	0.739	0.95	0.98	-0.53	-0.33	0.60
Item 28	424.9	348	0.003	1.22	1.09	2.74	1.23	0.49
Item 31	329.6	348	0.754	0.94	0.93	-0.68	-0.91	0.59
Item 36	318.1	348	0.873	0.91	0.92	-1.11	-1.20	0.65
Item 37	325.5	348	0.801	0.93	1.00	-0.51	0.04	0.55
Item 46	295.1	348	0.982	0.85	0.86	-1.98	-2.10	0.65
Item 47	318.7	348	0.868	0.91	0.96	-0.78	-0.56	0.64
Item 56	282.6	348	0.996	0.81	0.87	-1.88	-1.87	0.65
Item 60	293.8	348	0.984	0.84	0.89	-1.58	-1.67	0.65
Item 62	364.4	348	0.262	1.04	1.02	0.53	0.26	0.59
**WSS-CH—Parent**								
Item 14	370.7	339	0.114	1.09	0.94	0.78	-0.73	0.57
Item 15	273.0	339	0.996	0.80	0.84	-2.52	-2.33	0.65
Item 18	285.7	339	0.984	0.84	0.93	-1.44	-0.97	0.59
Item 19	317.3	339	0.796	0.93	0.93	-0.46	-0.88	0.56
Item 20	325.4	339	0.693	0.96	0.97	-0.41	-0.38	0.58
Item 22	321.2	339	0.748	0.95	0.97	-0.63	-0.48	0.60
Item 24	298.1	339	0.947	0.88	0.89	-1.69	-1.56	0.59
Item 29	355.0	339	0.265	1.04	1.01	0.57	0.10	0.52
Item 34	313.9	339	0.832	0.92	0.98	-0.94	-0.19	0.55
Item 38	334.2	339	0.563	0.98	0.96	-0.20	-0.51	0.54
Item 41	326.7	339	0.674	0.96	0.92	-0.49	-1.14	0.60
Item 52	381.3	339	0.056	1.12	1.01	1.40	0.20	0.53
Item 61	297.7	339	0.949	0.88	0.91	-1.55	-1.24	0.60
**WSS-CH—Organisational**								
Item 03	322.6	352	0.867	0.91	0.92	-1.00	-1.19	0.60
Item 04	340.1	352	0.665	0.96	0.98	-0.27	-0.28	0.60
Item 06	328.0	352	0.816	0.93	0.99	-0.76	-0.06	0.56
Item 10	406.1	352	0.024	1.15	1.11	2.07	1.58	0.42
Item 30	358.2	352	0.398	1.02	0.99	0.19	-0.09	0.56
Item 32	252.7	352	1.000	0.72	0.84	-2.95	-2.55	0.67
Item 39	291.6	352	0.992	0.83	0.86	-2.18	-2.20	0.64
Item 43	341.1	352	0.652	0.97	0.96	-0.34	-0.54	0.59
Item 45	356.2	352	0.427	1.01	1.01	0.14	0.11	0.52
Item 48	353.7	352	0.465	1.00	1.03	0.05	0.37	0.52
Item 53	313.3	352	0.932	0.89	0.92	-1.54	-1.27	0.60
Item 57	316.4	352	0.914	0.90	0.89	-1.38	-1.68	0.60

### Confirmatory factor analysis

After the RA, we analysed our data using a CFA. We tested four different models for each of the scales and goodness-of-fit statistics are presented in Table 7. The first model used all scale items and had modification indices examined. We qualitatively reviewed the error covariances and included in the second model the items deemed as theoretically plausible based on the Stage 4 focus groups. The third and fourth models used the same approach but removed the misfitting items, as suggested by the Rasch analysis, from the models. There were clear improvements to model fits on each iteration for the WRS-CH scale. This was similar for the WSS-CH scale, but despite models 2 and 4 showing virtually the same fit parameters, model 2 had slightly better fits.

**Table 7:** Goodness-of-fit statistics for confirmatory factor analysis models

	**Chi-squared**	**df**	*p* value	**CFI**	**TLI**	**RMSEA (95%CI)**	**SRMR**
WRS-CH model 1	1660.78	594	0.000	0.860	0.860	0.069(0.065–0.073)	0.109
WRS-CH model 2	1324.87	579	0.000	0.906	0.897	0.059(0.055–0.063)	0.096
WRS-CH model 3	1226.81	434	0.000	0.881	0.872	0.069(0.065–0.074)	0.101
WRS-CH model 4 (Final)	919.64	422	0.000	0.925	0.918	0.056(0.051–0.061)	0.086
WSS-CH model 1	3303.05	1826	0.000	0.916	0.913	0.051(0.048–0.054)	0.080
WSS-CH model 2	3114.12	1812	0.000	0.926	0.922	0.048(0.045–0.051)	0.077
WSS-CH model 3	1926.18	857	0.000	0.910	0.904	0.062(0.058–0.066)	0.079
WSS-CH model 4 (Final)	1833.92	851	0.000	0.916	0.911	0.060(0.056–0.063)	0.077

Model 1: All items from original scales

Model 2: All items from original scales and error covariances included

Model 3: Dropped items suggested by Rasch Analysis

Model 4: Dropped items suggested by Rasch Analysis and error covariances included

**Fig. 2:**
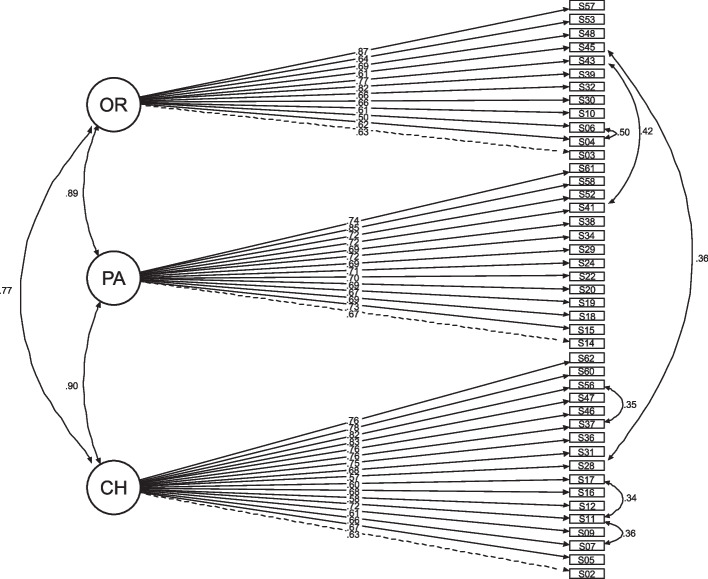
Results for the confirmatory factor analysis for the Work Rewards Scale – Children’s Hospices (WRS-CH). Model fit indices: Chi-squared: 919.64, df: 422, *p* value: 0.000, CFI: 0.93, TLI: 0.92, RMSEA (95%CI): 0.056(0.051–0.061), SRMR: 0.086

**Fig. 3:**
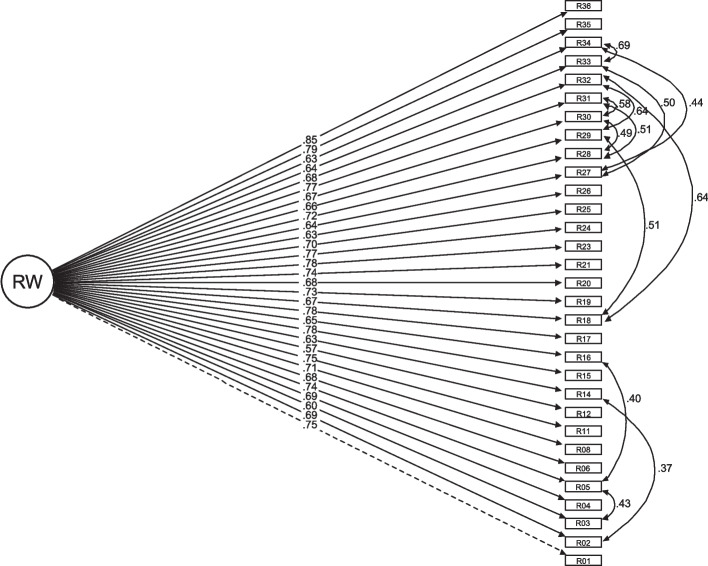
Results for the confirmatory factor analysis for the Work Stressors Scale – Children’s Hospices (WSS-CH). Model fit indices: Chi-squared: 1833.92, df: 851, *p* value: 0.000, CFI: 0.92, TLI: 0.92, RMSEA (95%CI): 0.060(0.056–0.063), SRMR: 0.077

The final one-factor structure for the WRS-CH scale and three-factor structure for the WSS-CH scale are presented in Figs. 2 and 3, respectively. Detailed unstandardised and standardised loadings and SE for both models are available on Supplementary Tables 6 and 7.

### Construct validity

We tested correlations between the WRS-CH scale and WSS-CH scale and Burnout and HSE Management standards (Table 8). The WRS-CH scale was negatively correlated and WSS-CH positively correlated with burnout. The WRS-CH scale was positively correlated, and the WSS-CH [Child] sub-scale was negatively correlated with all HSE dimensions, except Demands. The WSS-CH [Parent] sub-scale was negatively correlated with all HSE dimensions, except Peer support and Change. Finally, the WSS-CH [Organisational] sub-scale was negatively correlated with all HSE dimensions, except Change.

**Table 8:** Correlations between the new measures WRS-CH and WSS-CH and maslach burnout inventory Health and Safety Executive’s Management standards indicator tool (HSE)

	**Burnout**	**HSE Control**	**HES Managers support**	**HSE Peer support**	**HSE Relationships**	**HSE Demands**	**HSE Role**	**HSE Change**
WRS-CH—Rewards	-0.20^***^	0.18^***^	0.33^***^	0.33^***^	0.16^**^	0.01	0.21^***^	0.29^***^
WSS-CH—Child stressors	0.28^***^	-0.29^***^	-0.15^***^	-0.09^***^	-0.22^***^	-0.23	-0.1^***^	-0.10^***^
WSS-CH—Parent stressors	0.25^***^	-0.22^***^	-0.14^**^	-0.13	-0.24^***^	-0.30^***^	-0.17^**^	-0.08
WSS-CH—Organisation stressors	0.38^***^	-0.29^***^	-0.34^**^	-0.32^**^	-0.48^***^	-0.41^***^	-0.32^***^	-0.3

^***^p ≤ 0.001

^**^p ≤ 0.01

^*^p ≤ 0.05

The strongest correlation between the WSS-CH scale and burnout was with Organisation stressors (*r* = 0.38, *p* ≤ 0.001). The WSS-CH [Child] sub-scale had its strongest correlation with HSE Control (*r* = -0.29, *p* ≤ 0.001), WSS-CH [Parent] sub-scale with HSE Demands (*r* = -0.30, *p* ≤ 0.001); and WSS-CH [Organisational] sub-scale with HSE Relationships (*r* = -0.30, *p* ≤ 0.001); the strongest correlation between the WRS-CH scale and HSE was with manager’s support (*r* = 0.33, *p* ≤ 0.001) and peers’ support (*r* = 0.33, *p* ≤ 0.001).

## Discussion

This paper reports the development and validation of two new, robust scales to measure the work-related stressors and rewards of care staff working in UK children’s hospices. The Work-Related Rewards Scale – Children’s Hospices (WRS-CH) is a 31-item scale that provides a total score of the perceived intensity of the work-related rewards experienced by children’s hospice staff. The Work-Related Stressor Scale – Children’s Hospices (WSS-CH) is a 42-item scale that provides a total score of the perceived intensity of work-related stressors experienced by children’s hospice staff, as well as sub-scale scores for ‘Child’ (caring for an ill or dying child); ‘Parent’ (working with parents and families); and ‘Organisation’ (team conflict, workload and work environment sources of stresses).

The four stages of the development of the scales are presented in this paper. The first three stages highlighted important evidence on the relationship between the work-related stressors and rewards experienced by staff working in children’s hospices and those experienced by staff working in paediatric oncology. Version 4 of the WSS-CH and WRS-CH scales showed that there are many similarities between the two specialisms, with over 80% of the items in each scale being the same, as well as some key differences. One key difference between the paediatric oncology scales and the children hospice scales is that the death of a child in the former was universally characterised as a stressor [10], whereas it is present in both the stressors and rewards hospice scales. Thus, children’s hospice staff seem to be able to find rewards in the death of a patient, whereas staff working in paediatric oncology seem to consider the death of a child to be a failure. This is likely because of the different focus of these specialisms, with paediatric oncology focused on providing curative and palliative care, whereas hospices mainly provide palliative care [53, 54]. It is worth noting that the perceived rewards for hospice staff associated with caring for the child, and their family, at the end of life are heavily reliant on there being sufficient time and the necessary expertise to provide high quality care [55].

Just as in paediatric oncology, the work-related stressors in the children’s hospice settings are not centred around caring for the child or young person, and their family at the end of life, but are multi-dimensional, with organisational stressors such as feeling undervalued or unsupported by management included alongside children- and family-related factors.

The findings indicate the rewards for children’s hospice staff are not financial, nor are they about the terms and conditions of work. Instead, they focus on the rewarding nature of developing relationships with patients and their families, seeing positive outcomes for families, and working with colleagues who you respect and who make you feel respected. Developing and maintaining expertise through self-development also appears to be equally key. This matches the JD-R theory of working conditions, which notes the importance of working relationships, and personal growth and learning, in protecting against work-related stress [19].

In line with recommendations by others researching work-related stress [56–58], these findings highlight the importance of using context-specific measures to assess work-related stressors and rewards: within the field of children’s hospices, reliance on generic measures would provide an incomplete picture of staff experiences and could result in inappropriately targeted interventions.

Most of the items with poor fit, as determined by the RA and CFA, tended to be either those highlighted by staff in Stages 1–3 as having the most ambiguous meanings, or were items that had remained unchanged from the original scales (except items 18 and 32 from WRS-CH and item 17 from WSS-CH).

In addition, there was evidence that some items in CFA models had correlated errors. All suggested items were reviewed and decisions on error covariances to be specified in the models were checked for similarly worded items [40]. The final models showed better fits after including the error covariances. Despite the Rasch Analysis not showing clear support for removing these items from the scales, future studies may find that some of these may be less relevant depending on the context and professional compositions of the caring teams.

### Strengths and limitations

This study applied a rigorous stepwise approach to adapt the scales to the children’s’ hospice environment. This included using a mixed-methods approach with multiple analytical strategies to derive the final versions of the scale. The UK-wide survey (Stage 4) was based on a diverse sample of the hospice care team population in the UK.

Staff were surveyed during COVID-19 when approximately 40% of the sample was not following their normal working arrangements. At a time when the future of the hospice they worked for and their job security was uncertain, this may have influenced the stressors being experienced at the time. Future research could measure the scales’ test–retest reliability.

## Conclusion

This study developed two scales designed to assess work-related stressors and rewards for use with children’s hospice care teams by adapting scales developed in paediatric oncology settings. The new scales showed good psychometric properties with factor structures that are equal to the original measures. The new scales can be useful in clinical practice and research to assess work-related perceived intensity of the work-related rewards and stressors by children’s hospice staff, and are available for use in an online repository [59].

### Supplementary Information

**Additional file 1:** **Supplementary Table 1. **Items list for Work Rewards Scale – Children’s Hospices scale (WRS-CH).** Supplementary Table 2. **Items list for WSS-CH: Work Stressors Scale – Children’s Hospices scale (WSS-CH).** Supplementary Table 3. **Rewording of WSS-PO to WSS-CH.** Supplementary Table 4. **Rewording of WRS-PO to WRS-CH.** Supplementary Table 5. **Overall model fit statistics for the model using all scale items (preliminary models).** Supplementary Table 6. **Final model unstandardised and standardised loadings and standard errors for Work Rewards Scale – Children’s Hospices (WRS-CH). **Supplementary Table 7. **Final model unstandardised and standardised loadings and standard errors for Work Stressors Scale – Children’s Hospices (WSS-CH).

## Data Availability

The datasets generated and analysed for this study are available from the corresponding author on reasonable request.
