# Supplementary material for: A new scale assessing the stressors and rewards of children’s hospice work
Source: BMC Palliat Care. 2023 Sep 13;22:136. doi: 10.1186/s12904-023-01246-w (PMC10498554; doi:10.1186/s12904-023-01246-w)
Supplement: Supplementary file 1 — Additional file 1: Supplementary Table 1. Items list for Work Rewards Scale – Children’s Hospices scale (WRS-CH). Supplementary Table 2. Items list for WSS-CH: Work Stressors Scale – Children’s Hospices scale (WSS-CH). Supplementary Table 3. Rewording of WSS-PO to WSS-CH. Supplementary Table 4. Rewording of WRS-PO to WRS-CH. Supplementary Table 5. Overall model fit statistics for the model using all scale items (preliminary models). Supplementary Table 6. Final model unstandardised and standardised loadings and standard errors for Work Rewards Scale – Children’s Hospices (WRS-CH). Supplementary Table 7. Final model unstandardised and standardised loadings and standard errors for Work Stressors Scale – Children’s Hospices (WSS-CH). [file 12904_2023_1246_MOESM1_ESM.docx]

Supplementary Table 1 Items list for Work Rewards Scale – Children’s Hospices scale (WRS-CH)

| 1 - Feeling that I’ve made a difference to a child |
| --- |
| 2 - Knowing how to help |
| 3 - Knowing that we are providing a good service |
| 4 - Helping a child cope with their situation |
| 5 - Knowing that I am doing something really useful |
| 6 - Seeing a child having fun |
| 7 - Having a chance to develop my role^ⱡ^ |
| 8 - Families valuing my opinion |
| 9 - Doing something that makes a child feel better^ⱡ^ |
| 10 - Feeling I’m respected by other members of the team^ⱡ^ |
| 11 - Getting thanks from parents |
| 12 - Knowing that other members of staff need my help |
| 13 - Being able to work flexibly in my role^ⱡ^ |
| 14 - Being able to identify what I can do about a problem |
| 15 - Seeing a family develop the ability to cope |
| 16 - The feeling that I’m doing my job well |
| 17 - Sharing the high points with a child and their family |
| 18 - Getting it right for a child when they die* |
| 19 - Supporting a sibling or other family member* |
| 20 - Working with people who enjoy the same sort of work as me |
| 21 - Being involved in both the clinical and psychosocial aspects of the child's care |
| 22 - Knowing I’m making the situation easier for families and for children^ⱡ^ |
| 23 - Being able to get a child to interact with me |
| 24 - A family getting to know me |
| 25 - Other staff appreciating my contribution to a child's care |
| 26 - Developing new skills and gaining knowledge |
| 27 - Feeling that I am working in an expert team |
| 28 - Being one of the people that parents and siblings feel they can open up to |
| 29 - Supporting the family after a child has died |
| 30 - Developing long-lasting relationships with families |
| 31 - Families are pleased to see me |
| 32 - Making a child comfortable at the end of life* |
| 33 - Working in a supportive team |
| 34 - Working in a team that is committed to the children |
| 35 - Empowering children and their families to make decisions about care* |
| 36 - Helping children and their families to make memories* |

* Items not in the original scale
^ⱡ^ Items dropped in the final version of the scales (7, 9, 10, 13, 22).

Supplementary Table 2 Items list for WSS-CH: Work Stressors Scale – Children’s Hospices scale (WSS-CH)

| \| **WSS-CH - Child Sub-Scale items** \| **WSS-CH - Parent Sub-Scale items** \| **WSS-CH - Organisational Sub-Scale items** \| \| --- \| --- \| --- \| \| 1 - Working in an environment where there’s lots of stress, sadness and anxiety ^ⱡ^ \| 14 - Families becoming dependent on me \| 3 - Other members of the care team not responding to my requests for help \| \| 2 - Having to answer parents’ questions during the end of life stage \| 15 - Not feeling sure how much parents have understood what I’ve told them \| 4 - Other staff being quick to find fault with me \| \| 5 - Talking to children about distressing subjects \| 18 - Parents expecting me to do things that are not part of my job \| 6 - Feeling my skills are not recognised \| \| 7 - Watching a child deteriorate day-by-day \| 19 - A parent not letting me talk to their child alone \| 8 - Working to targets set by managers ^ⱡ^ \| \| 9 - A parent getting upset \| 20 - Parents not supporting what I’m doing with their child \| 10 - Difficulties liaising with other services \| \| 11 - When a child deteriorates very quickly \| 22 - Families being aggressive towards other members of the care team \| 25 - Staff being obstructive ^ⱡ^ \| \| 12 - Containing my emotions after the death of a child \| 24 - Caring for a family who is in desperate social circumstances \| 26 - Not being able to do my job to the standard I would like ^ⱡ^ \| \| 13 - Not feeling confident when caring for a child* ^ⱡ^ \| 27 - A parent not being happy with the care I am providing ^ⱡ^ \| 30 - Feeling my work is being closely monitored by managers \| \| 16 - Not knowing after a child dies whether we chose the right approach \| 29 - Having to be positive with a family when I know the outcome will not be good \| 32 - Managers placing unfair demands on me \| \| 17 - Seeing a child's body deteriorate after death* \| 34 - Supporting parents who feel guilty about treatment decisions \| 35 - Coping with funding pressures* ^ⱡ^ \| \| 21 - Periods when I see a lot of children who are at the end of life ^ⱡ^ \| 38 - Dealing with parents who are in conflict with each other \| 39 - Not having time when a family asks for help \| \| 23 - Dealing with a lot of deaths in a short space of time ^ⱡ^ \| 40 - Having to respect a parent’s wishes when I don’t agree with them ^ⱡ^ \| 42 - Feeling that members of the team don’t value each other’s opinion ^ⱡ^ \| \| 28 - Lots of very complex children at the same time \| 41 - Not agreeing with how a parent is behaving towards their child \| 43 - Feeling that a child's care is being compromised \| \| 31 - Supporting the family after the death of their child \| 49 - A family expecting things to be done within an unrealistic timeframe ^ⱡ^ \| 44 - Being interrupted when I am talking to a family ^ⱡ^ \| \| 33 - Giving a parent bad news ^ⱡ^ \| 52 - Difficulty establishing a relationship with a family \| 45 - Being expected to take on a wide variety of tasks \| \| 36 - The sudden death of a child* \| 55 - A parent asking me what I would do in their situation ^ⱡ^ \| 48 - Not having a quiet space available \| \| 37 - Being involved in telling parents their child’s diagnosis \| 58 - A parent demanding more and more support from me ^ⱡ^ \| 50 - Staff not passing on the information I need to do my job ^ⱡ^ \| \| 46 - A prolonged death \| 61 - Parents not understanding the importance of adhering to medical regimes \| 51 - Finding that I don’t switch off properly after I have finished work ^ⱡ^ \| \| 47 - Feeling responsible for a child’s distress \|  \| 53 - Working with a team member who isn’t pulling their weight \| \| 56 - Being involved in a consultation where parents are told the prognosis is worse than we thought \|  \| 54 - Having to adapt what I say to parents to cater for their level of understanding ^ⱡ^ \| \| 60 - Being responsible for controlling the symptoms of a dying child \|  \| 57 - Disagreements about how best to care for a child* \| \| 62 - Feeling like everything I do can have major consequences for a child \|  \| 59 - Feeling that I can’t keep up with requests from other team members ^ⱡ^ \|   * Items not in the original scale  ^ⱡ^ Items dropped in the final version of the scale (1, 8, 13, 21, 23, 25, 26, 27, 33, 35, 40, 42, 44, 49, 50, 51, 54, 55, 58, 59) |
| --- | --- | --- | --- | --- | --- | --- | --- | --- | --- | --- | --- | --- | --- | --- | --- | --- | --- | --- | --- | --- | --- | --- | --- | --- | --- | --- | --- | --- | --- | --- | --- | --- | --- | --- | --- | --- | --- | --- | --- | --- | --- | --- | --- | --- | --- | --- | --- | --- | --- | --- | --- | --- | --- | --- | --- | --- | --- | --- | --- | --- | --- | --- | --- | --- | --- | --- | --- | --- | --- |

Supplementary Table 3 Rewording of WSS-PO to WSS-CH

| **Type** | **Wording in original** | **Wording after focus groups** | **Wording after cognitive interviews** |
| --- | --- | --- | --- |
| Reword | A protracted death | A prolonged death | 46 – A prolonged death |
| Reword | Working in an environment that is very noisy | Not having a quiet space available | 48 – Not having a quiet space available |
| Reword | Periods when I only see patients who are ill | Periods when I only see children who are at the end of life | 21 – Periods when I see a lot of children who are at the end of life |
| Reword | Lots of very sick children on the ward at once | Lots of very complex children at the same time | 28 – Lots of very complex children at the same time |
| Reword | Other departments not prioritising our patients | Difficulties liaising with other services | 10 – Difficulties liaising with other services |
| New | N/A | Coping with funding pressures | 35 – Coping with funding pressures |
| New | N/A | The unexpected death of a child | 36 – The sudden death of a child |
| New | N/A | Responding to the urgent care needs of a child | REMOVED |
| New | N/A | A traumatic death | REMOVED |
| New | N/A | Disagreements about best to care for a child | 57 – Disagreements about best to care for a child |
| New | N/A | Not feeling confident when caring for a child | 13 – Not feeling confident when caring for a child |
| New | N/A | Not feeling competent when caring for a child | REMOVED |
| New | N/A | Seeing a child's body deteriorate | 17 – Seeing a child's body deteriorate after death |
| Original | Watching a child deteriorate day-by-day | Watching a child deteriorate day-by-day | 7 – Watching a child deteriorate day-by-day |
| Original | Staff being obstructive | Staff being obstructive | 25 – Staff being obstructive |
| Original | Other staff being quick to find fault with me | Other staff being quick to find fault with me | 4 – Other staff being quick to find fault with me |
| Original | Feeling parents are being pushed into agreeing to treatments | REMOVED | N/A |
| Original | Not knowing after a child dies if we chose the wrong approach | Not knowing after a child dies if we chose the wrong approach | 16 – Not knowing after a child dies if we chose the right approach |
| Original | Parents expecting me to do things that are not part of my job | Parents expecting me to do things that are not part of my job | 18 – Parents expecting me to do things that are not part of my job |
| Original | Parents thinking they know better than me | Parents thinking they know better than me | REMOVED |
| Original | Having a family on my caseload who is in desperate social circumstances | Having a family on my caseload who is in desperate social circumstances | 24 – Caring for a family who is in desperate social circumstances |
| Original | Senior managers placing unfair demands on me | Senior managers placing unfair demands on me | 32 – Managers placing unfair demands on me |

**Supplementary Table 4 Rewording of WRS-PO to WRS-CH**

| **Type** | **Wording in original** | **Wording after focus groups** | **Wording after cognitive interviews** |
| --- | --- | --- | --- |
| Original | Being involved in both the medical and psychosocial aspects of the child's care | Being involved in both the medical and psychosocial aspects of the child's care | 21 – Being involved in both the clinical and psychosocial aspects of the child's care |
| Original | Seeing a child at follow up when they're well and thriving | Seeing a child having fun | 6 – Seeing a child having fun |
| Original | Feeling I've contributed to a child returning to a normal life | REMOVED | N/A |
| Original | Seeing a child get better | REMOVED | N/A |
| Original | Seeing a patient further down the line | REMOVED | N/A |
| Original | Seeing a family move on with their lives | REMOVED | N/A |
| New | N/A | Supporting a child to have a good death | 18 – Getting it right for a child when they die |
| New | N/A | Supporting other family members | 19 – Supporting a sibling or other family member |
| New | N/A | Supporting the family after a child has died | 29 – Supporting the family after a child has died |
| New | N/A | N/A | 32 – Making a child comfortable at the end of life |
| New | N/A | Empowering children and their families to make decisions about care | 35 – Empowering children and their families to make decisions about care |
| New | N/A | Helping families to make memories | 36 – Helping children and their families to make memories |
| Reworded | Being involved ... from the beginning to the end of their treatment | Being involved ... from the beginning to the end of the service | REMOVED |
| Reworded | Being able to work creatively in my role | Being able to work creatively in my role | 13 – Being able to work flexibly in my role |

Supplementary Table 5 Overall model fit statistics for the model using all scale items (preliminary models)

|  | **Cronbach's  alpha** | **Unidimensionality** | | **Overall goodness-of-fit.** | |
| --- | --- | --- | --- | --- | --- |
|  |  | LR-test  (Chi-squared df) | p value | LR-test  (Chi-squared df) | p value |
| WRS-CH (All items model) | 0.94 | 339.23 (1279) | 1.00 | 32.13 (25) | 0.154 |
| WSS-CH (All items model) - Child | 0.93 | 337.89 (479) | 1.00 | 79.95 (41) | 0.000 |
| WSS-CH (All items model) - Parent | 0.92 | 181.38 (319) | 1.00 | 75.83 (33) | 0.000 |
| WSS-CH (All items model) - Organisational | 0.92 | 272.96 (483) | 1.00 | 117.64 (41) | 0.000 |

Cronbach's alpha. Should be > 0.70 to be statistically reliable.

Unidimensionality tested using Martin-Löf-Test. Non-significant p-value at the 5% level supports unidimensionality.

Overall goodness-of-fit tested using Andersen's likelihood-ratio test. Non-significant p-value at the 5% level supports a good fit.

WRS-CH: Work Rewards Scale – Children’s Hospices

WSS-CH: Work Stressors Scale – Children’s Hospices

Supplementary Table 6 Final model unstandardised and standardised loadings and standard errors for Work Rewards Scale – Children’s Hospices (WRS-CH)

| Item | Unstandardised Loading | Unstandardised SE | Standardised Loading | Standardised SE |
| --- | --- | --- | --- | --- |
| Item 01 | 1.00 | 0.00 | 0.75 | 0.04 |
| Item 02 | 0.91 | 0.07 | 0.69 | 0.05 |
| Item 03 | 0.80 | 0.06 | 0.60 | 0.05 |
| Item 04 | 0.92 | 0.07 | 0.69 | 0.04 |
| Item 05 | 0.98 | 0.06 | 0.74 | 0.04 |
| Item 06 | 0.91 | 0.09 | 0.69 | 0.06 |
| Item 08 | 0.94 | 0.07 | 0.71 | 0.04 |
| Item 11 | 1.00 | 0.07 | 0.75 | 0.03 |
| Item 12 | 0.76 | 0.07 | 0.57 | 0.04 |
| Item 14 | 0.84 | 0.06 | 0.63 | 0.04 |
| Item 15 | 1.04 | 0.07 | 0.79 | 0.03 |
| Item 16 | 0.87 | 0.07 | 0.65 | 0.04 |
| Item 17 | 1.04 | 0.07 | 0.78 | 0.03 |
| Item 18 | 0.88 | 0.07 | 0.67 | 0.05 |
| Item 19 | 0.97 | 0.07 | 0.73 | 0.04 |
| Item 20 | 0.91 | 0.07 | 0.68 | 0.05 |
| Item 21 | 0.98 | 0.07 | 0.74 | 0.03 |
| Item 23 | 1.03 | 0.07 | 0.78 | 0.04 |
| Item 24 | 1.02 | 0.07 | 0.77 | 0.03 |
| Item 25 | 0.93 | 0.06 | 0.70 | 0.04 |
| Item 26 | 0.84 | 0.07 | 0.63 | 0.04 |
| Item 27 | 0.85 | 0.07 | 0.64 | 0.04 |
| Item 28 | 0.96 | 0.07 | 0.72 | 0.04 |
| Item 29 | 0.87 | 0.07 | 0.66 | 0.04 |
| Item 30 | 0.90 | 0.07 | 0.67 | 0.04 |
| Item 31 | 1.02 | 0.07 | 0.77 | 0.04 |
| Item 32 | 0.90 | 0.08 | 0.68 | 0.04 |
| Item 33 | 0.85 | 0.07 | 0.64 | 0.05 |
| Item 34 | 0.83 | 0.08 | 0.63 | 0.06 |
| Item 35 | 1.05 | 0.07 | 0.79 | 0.03 |
| Item 36 | 1.13 | 0.07 | 0.85 | 0.04 |

Supplementary Table 7 Final model unstandardised and standardised loadings and standard errors for Work Stressors Scale – Children’s Hospices (WSS-CH)

| Item | Unstandardised Loading | Unstandardised SE | Standardised Loading | Standardised SE |
| --- | --- | --- | --- | --- |
| **WSS-CH - Child** |  |  |  |  |
| Item 02 | 1.00 | 0.00 | 0.63 | 0.04 |
| Item 05 | 1.07 | 0.07 | 0.67 | 0.04 |
| Item 07 | 1.06 | 0.07 | 0.66 | 0.04 |
| Item 09 | 0.97 | 0.08 | 0.61 | 0.04 |
| Item 11 | 1.14 | 0.08 | 0.72 | 0.03 |
| Item 12 | 0.92 | 0.07 | 0.58 | 0.04 |
| Item 16 | 1.09 | 0.08 | 0.68 | 0.04 |
| Item 17 | 0.96 | 0.08 | 0.60 | 0.04 |
| Item 28 | 0.90 | 0.08 | 0.57 | 0.05 |
| Item 31 | 1.08 | 0.08 | 0.68 | 0.04 |
| Item 36 | 1.20 | 0.08 | 0.75 | 0.03 |
| Item 37 | 1.22 | 0.09 | 0.76 | 0.04 |
| Item 46 | 1.21 | 0.08 | 0.76 | 0.03 |
| Item 47 | 1.32 | 0.08 | 0.83 | 0.03 |
| Item 56 | 1.30 | 0.09 | 0.82 | 0.03 |
| Item 60 | 1.24 | 0.08 | 0.78 | 0.03 |
| Item 62 | 1.21 | 0.09 | 0.76 | 0.03 |
| **WSS-CH - Parent** |  |  |  |  |
| Item 14 | 1.00 | 0.00 | 0.68 | 0.04 |
| Item 15 | 1.08 | 0.07 | 0.73 | 0.04 |
| Item 18 | 1.02 | 0.07 | 0.69 | 0.04 |
| Item 19 | 0.99 | 0.07 | 0.67 | 0.05 |
| Item 20 | 1.02 | 0.08 | 0.69 | 0.04 |
| Item 22 | 1.04 | 0.08 | 0.70 | 0.03 |
| Item 24 | 1.06 | 0.07 | 0.71 | 0.03 |
| Item 29 | 1.02 | 0.08 | 0.69 | 0.03 |
| Item 34 | 1.07 | 0.07 | 0.72 | 0.03 |
| Item 38 | 1.02 | 0.08 | 0.69 | 0.04 |
| Item 41 | 1.06 | 0.08 | 0.72 | 0.03 |
| Item 52 | 1.07 | 0.08 | 0.72 | 0.04 |
| Item 58 | 1.27 | 0.08 | 0.85 | 0.02 |
| Item 61 | 1.09 | 0.07 | 0.74 | 0.03 |
| **WSS-CH – Organisational** |  |  |  |  |
| Item 03 | 1.00 | 0.00 | 0.63 | 0.04 |
| Item 04 | 0.98 | 0.08 | 0.62 | 0.05 |
| Item 06 | 0.80 | 0.08 | 0.51 | 0.05 |
| Item 10 | 0.96 | 0.09 | 0.61 | 0.05 |
| Item 30 | 1.04 | 0.09 | 0.66 | 0.04 |
| Item 32 | 1.04 | 0.09 | 0.66 | 0.04 |
| Item 39 | 1.29 | 0.10 | 0.82 | 0.03 |
| Item 43 | 1.21 | 0.09 | 0.77 | 0.03 |
| Item 45 | 0.95 | 0.09 | 0.61 | 0.05 |
| Item 48 | 1.09 | 0.10 | 0.69 | 0.04 |
| Item 53 | 1.01 | 0.08 | 0.64 | 0.04 |
| Item 57 | 1.37 | 0.10 | 0.87 | 0.03 |
